# Comorbidity of CRHR2 gene variants in type 2 diabetes and depression

**DOI:** 10.1192/j.eurpsy.2022.1050

**Published:** 2022-09-01

**Authors:** M. Amin, J. Ott, R. Wu, T. Postolache, M. Vergare, C. Gragnoli

**Affiliations:** 1 Faculty of Medicine, University of Khartoum, Department Of Biochemistry And Molecular Biology, Khartoum, Sudan; 2 Rockefeller University, Laboratory Of Statistical Genetics, New York, United States of America; 3 Penn State College of Medicine, Department Of Public Health Sciences, Hershey, United States of America; 4 Penn State College of Medicine, Departments Of Statistics, Hershey, United States of America; 5 University of Maryland School of Medicine, Mood And Anxiety Program, Department Of Psychiatry, Baltimore, United States of America; 6 Veterans Integrated Service Network (VISN) 5, VA Capitol Health Care Network, Rocky Mountain Mental Illness Research Education And Clinical Center (mirecc), Veterans Integrated Service Network (visn), Denver, United States of America; 7 Veterans Integrated Service Network (VISN) 5, VA Capitol Health Care Network, Mental Illness Research Education And Clinical Center (mirecc), Baltimore, United States of America; 8 Sidney Kimmel Medical College, Thomas Jefferson University, Department Of Psychiatry And Human Behavior, Philadelphia, United States of America; 9 Creighton University School of Medicine, Division Of Endocrinology, Department Of Medicine, Philadelphia, United States of America; 10 Bios Biotech Multi-Diagnostic Health Center, Molecular Biology Laboratory, Rome, Italy

**Keywords:** Type 2 diabetes, Depression, CRHR2, MDD

## Abstract

**Introduction:**

The corticotropin-releasing hormone receptor 2 (CRHR2) gene encodes CRHR2, which is an important element in the hypothalamic-pituitary-adrenal physiologic response towards stress culminating in hyperglycemia, insulin resistance, mood disorders and depression (MDD). CRHR2-/- mice are hypersensitive to stress, and the CRHR2 locus in humans has been linked to type 2 diabetes (T2D) and MDD.

**Objectives:**

Several variants in the CRHR2 gene have been reported in patients with bipolar disorder, post-traumatic stress disorder, and T2D, but variants in the gene have not been investigated in families with T2D and MDD.

**Methods:**

We genotyped 212 Italian families with T2D and MDD. We tested 17 SNPs in the CRHR2 gene using two-point parametric-linkage and linkage-disequilibrium (LD) analysis with the following models: dominant with complete-penetrance (D1), dominant with incomplete-penetrance (D2), recessive with complete-penetrance (R1) and recessive with incomplete-penetrance (R2).

**Results:**

We detected linkage to and/or LD with: MDD for 3 SNPs/D1, 2 SNPs/D2, 3 SNPs/R1, and 3 SNPs/R2; and, T2D for 3 SNPs/D1, 2 SNPs/D2, 2 SNPs/R1 and 1 SNP/R2. Two independent SNPs were comorbid. Interestingly, the variants linked to or in LD with MDD had in general higher statistical significance level than the variants linked to T2D, despite that the families were primarily ascertained for T2D.

**Conclusions:**

Our study shows for the first time that the CRHR2 gene which encodes CRHR2 is in linkage to and linkage disequilibrium with MDD and T2D, thereby contributing, in families with T2D, to both disorders and underlying the shared genetic pathogenesis of their comorbidity

**Disclosure:**

No significant relationships.

